# Antimicrobial Stewardship Impact on Antibiotic Use in Three Tertiary Hospitals in Zambia: A Comparative Point Prevalence Survey

**DOI:** 10.3390/antibiotics14030284

**Published:** 2025-03-10

**Authors:** Steward Mudenda, Kenneth Kapolowe, Uchizi Chirwa, Melvin Chanda, Raphael Chanda, Rodney Kalaba, Sombo Fwoloshi, Christabel Phiri, Mukuka Mwamba, Robert Kajaba Chirwa, Kotey Nikoi, Linda Musonda, Kaunda Yamba, Josepsh Yamweka Chizimu, Chitalu Chanda, Tamica Mubanga, Chisha Simutowe, John Kasanga, Mulope Mukanwa, Katongo Hope Mutengo, Philip Matthew, Fabian Maza Arnedo, Jyoti Joshi, Jonathan Mayito, Ruth Nakazwe, Maisa Kasanga, Duncan Chanda

**Affiliations:** 1 Department of Pharmacy, School of Health Sciences, University of Zambia, Lusaka 10101, Zambia; 2 University Teaching Hospitals, Lusaka 10101, Zambia; kennethkapolowe2020@gmail.com (K.K.); uchola5@gmail.com (U.C.); chandamelvin@yahoo.com (M.C.); nraphaelchanda@gmail.com (R.C.); kourtnikate@gmail.com (R.K.); sombofwoloshi@gmail.com (S.F.); chigweyassin@gmail.com (C.P.); mukukamwamba@outlook.com (M.M.); bobchirwa@gmail.com (R.K.C.); koteyn@gmail.com (K.N.); musonda.lamba@reactafrica.org (L.M.); ruthnakazwe@yahoo.com (R.N.); kasangaanita@gmail.com (M.K.); 3 Antimicrobial Resistance Coordinating Committee, Zambia National Public Health Institute, Lusaka 10101, Zambia; kaundayamba@gmail.com (K.Y.); chizimuyjoseph@yahoo.com (J.Y.C.); 4 Ndola Teaching Hospital, Ndola 10101, Zambia; chtalu@gmail.com (C.C.); tamicamubanga@gmail.com (T.M.); bestsimutowe@gmail.com (C.S.); 5 Livingstone University Teaching Hospital, Livingstone 10101, Zambia; kasanga95.kj@gmail.com (J.K.); mukanwa.mulope@gmail.com (M.M.); katmutengo@yahoo.com (K.H.M.); 6 International Center for Antimicrobial Resistance Solutions (ICARS), Ørestads Boulevard 5, 2300 Copenhagen, Denmark; phima@icars-global.org (P.M.); fama@icars-global.org (F.M.A.); jyoti@icars-global.org (J.J.); joma@icars-global.org (J.M.); 7 Department of Epidemiology and Biostatistics, School of Public Health, Zhengzhou University, Zhengzhou 450001, China

**Keywords:** antimicrobial stewardship, impact, interventions, point prevalence survey, Zambia

## Abstract

**Introduction:** Antimicrobial stewardship (AMS) can improve the rational use of antibiotics in hospitals. This study assessed the impact of a multifaceted AMS intervention on antibiotic use and prescribing patterns at three tertiary hospitals in Zambia. **Methods:** Point Prevalence Surveys (PPS) were conducted in three tertiary hospitals in August 2022 and in October 2023. It was part of a 3-year AMS demonstration project that aimed to optimize the use of antibiotics in treating urinary tract infections (UTIs) and bloodstream infections (BSIs) in various health sector settings in Zambia. Up to 170 medical records in 2022 and 265 in 2023 were included in the assessment. **Results:** Overall, the prevalence of antibiotic use in this PPS was 75%. Eighty-one percent (81%) and 71% of patients assessed were on at least one antibiotic in 2022 and 2023, respectively, indicating a decrease of 10%. Similarly, prescribing ceftriaxone, the most prescribed antibiotic, declined from an average of 48% in 2022 to 38% in 2023. Adherence to Standard Treatment Guidelines (STGs) slightly increased from 42% in 2022 to 45% in 2023. Additionally, antibiotic prescribing was reduced from 1.38 to 1.21. **Conclusions:** Antimicrobial stewardship had an early positive impact on antibiotic use and adherence to Standard Treatment Guidelines.

## 1. Introduction

The discovery of antibiotics was a great milestone that transformed the treatment of infectious diseases [1,2]. It enabled the treatment of potentially fatal infections and allowed for the execution of intricate medical procedures with lower infectious morbidity [3,4]. However, over the years, antibiotics have been used indiscriminately, with over 50% of the patients receiving unnecessary antibiotics [5,6,7,8]. Additionally, the inappropriate use of antibiotics has led to the emergence and spread of antimicrobial resistance (AMR) [9,10,11]. AMR is a threat to global health and has had a negative impact on the economy and food insecurity as well as treatment failure and increased morbidity and mortality [9,12,13,14,15,16]. If not addressed, it is predicted that more than 10 million people will die annually by the year 2050 [17,18]. Due to these impacts of AMR, there is a need to instigate strategies such as antimicrobial stewardship (AMS) programs to address its emergence and spread [18,19,20,21,22,23].

AMR is a natural phenomenon but is accelerated by the indiscriminate use of antibiotics in humans, animals, agriculture, and the environment [18,24]. Some of the critical drivers of AMR include the overuse, underuse, and misuse of antibiotics in hospitals [25,26,27], compounded by the lack of awareness and non-adherence to the recommended treatment guidelines [28,29,30,31,32]. Further, AMR is also influenced by the lack of diagnostic services in healthcare facilities, especially in low- and middle-income countries (LMICs), which leads to the inappropriate use of antibiotics [33,34,35,36,37,38]. The risk of drug-resistant infections is high in healthcare facilities with low awareness and knowledge as well as a lack of training in antimicrobial use (AMU), AMR, and AMS [27,39,40,41].

Antimicrobial stewardship (AMS) programs are essential in improving the appropriate use of antibiotics to control AMR [42,43,44,45] through educating and training health workers on treatment guidelines, causes of AMR, rational use of antibiotics, and strategies to address AMR [19,43,46,47,48,49]. To ensure a standardized AMR response, in 2015, the WHO developed the Global Action Plan (GAP) to address AMR through a One Health approach to surveillance [50,51,52,53]. Member states of the WHO were encouraged to develop National Action Plans (NAP) on AMR to address the global AMR threat [51,54]. Additionally, in 2017, the WHO developed and implemented the Access, Watch, and Reserve (AWaRe) classification of antibiotics as an AMS tool to promote the rational prescribing of antibiotics to optimize antibiotic use [55,56,57,58,59,60,61]. The AWaRe classification of antibiotics includes the Access group antibiotics that must be available at all times in healthcare facilities, have fewer side effects and lower potential for the selection of AMR, and are recommended for empiric treatment of most common infections [58,61,62]. Further, the tool contains the Watch group antibiotics that have a higher potential for the selection of AMR and are usually used in sicker hospitalized patients and need monitoring to avoid their overuse [59,61,63]. Furthermore, the tool contains the Reserve group antibiotics that are reserved for the treatment of severe infections caused by multidrug-resistant microbes [59,61,63]. The AWaRe framework recommends that 60% of the antibiotic prescriptions must emanate from the Access group [55,57,58,64]. The 2024 United Nations General Assembly (UNGA) revised this indicator and recommended that hospitals should be prescribing at least 70% of Access group antibiotics [65]. Therefore, the AWaRe classification is a useful tool for monitoring antibiotic consumption, defining targets, and monitoring the effects of stewardship policies that aim to optimize antibiotic use and curb AMR [57,60,66]. Prescribers also need to adhere to the Standard Treatment Guidelines (STGs) and Essential Medicines List (EML) to prevent adverse outcomes on patients and AMR [31,67].

The use of antibiotics in hospitals must be monitored frequently, especially in the sub-Saharan African region, with reports of high use of antibiotics of more than 50% [68,69]. Point prevalence surveys (PPS) have become useful in monitoring the use of antibiotics, adherence to treatment guidelines, and estimation of the prevalence of infections among in-patients [70,71,72]. The WHO and Global PPS methods have been used in many countries to monitor the use of antibiotics in hospitals and policy development to address the irrational use of antibiotics [73,74,75,76,77,78,79,80,81]. For instance, a PPS in Pakistan found that 77.6% of in-patients were treated with antibiotics, and most had gynecological, gastrointestinal, and lower respiratory tract infections [82]. Another study in Kenya found that 67.7% of in-patients were treated with antibiotics, mostly as medical prophylaxis after delivery [83]. These findings and others reported in similar studies show a high prevalence of antibiotic use [5,82,83,84,85], which needs to be addressed.

In Zambia, studies have reported a high prevalence of antibiotic use of up to 99.2% [7,86,87,88,89,90,91,92,93,94] and there is evidence of a high prevalence of AMR above 80% [95,96,97,98,99,100,101,102,103,104,105,106]. Further, a global PPS conducted in Uganda, Ghana, Zambia, and Tanzania found a 50% overall prevalence of antibiotic use [107], while another in Zambia found that 59% of in-patients were on antibiotics, the majority of which was ceftriaxone prescribed for empirical use [90]. However, no study has evaluated the impact of implementing AMS programs on antibiotic use in hospitals. This study therefore assessed the impact of multifaceted AMS interventions on antibiotic use and prescribing patterns in three tertiary hospitals in Zambia. The study also assessed the prevalence of antibiotic use as per the WHO AWaRe classification of antibiotics. This study was part of a 3-year AMS demonstration project led by the University Teaching Hospital (UTH) AMS team and funded by the International Center for Antimicrobial Resistance Solutions (ICARS).

## 2. Results

Table 1 displays the findings from two separate surveys conducted in 2022 and 2023. The median ages (i.e., 45 years for 2022 and 44 for 2023) of surveyed patients and the variation in ages (IQR for 2022: 31, 60; IQR for 2023: 29, 56) of the two samples were insignificant.

The overall combined sample and that disaggregated per survey year were almost uniformly distributed between males (54%) and females (46%), though males were slightly more (Table 1). Compared to the other two sites, LTH had a small sample in 2022. There was insufficient evidence of an association between gender and survey year (*p* > 0.05).

This study found an overall compliance with the national Standard Treatment Guidelines (STGs) at 44%. Table 2 shows that there was very strong evidence of an association (*p*-value < 0.001) between compliance status with national STGs and survey year, indicating better compliance in 2023 compared to 2022. Though the overall proportion of compliant prescriptions did not appear to change by much from 2022 (42%) to 2023 (45%), there was an obvious reduction in the proportion of prescriptions in which the compliance was not assessable, with 39% and 7.6% in 2022 and 2023, respectively. Of the three sites, LTH reported the greatest positive change in the compliant proportion from 0% to 88%, while NTH showed a reduction from 59% to 22%. Like LTH, UTH showed a positive change from 37% to 46% compliant prescriptions.

According to Table 2, though the overall data showed evidence of a strong relationship (*p*-value = 0.001) between patients categorized by number of prescribed antibiotics and the survey year, only NTH showed statistical significance (*p*-value = 0.001). However, all sites generally showed a reduction in the number and proportion of prescriptions with 3 or more antibiotics in 2023 compared to 2022. Overall, since 25% of patients were not on antibiotics, the prevalence of antibiotic use was 75% (Table 2).

**Figure 1:**
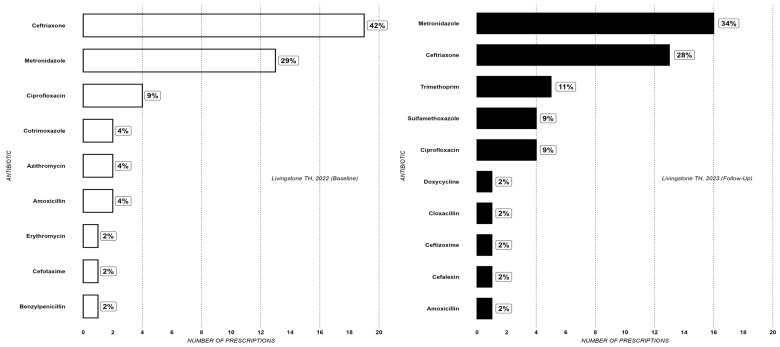
Antibiotic prescribing rate at Livingstone TH at baseline (2022) and follow-up (2023).

Figure 1 illustrates the finding that ceftriaxone and metronidazole were the most prescribed antibiotics at LTH in both surveys. Together they accounted for about 71% in 2022 and 62% of all prescribed antibiotics. The reduction was primarily due to a change in the proportion accounted for by ceftriaxone from 42% to 39% (Figure 1).

**Figure 2:**
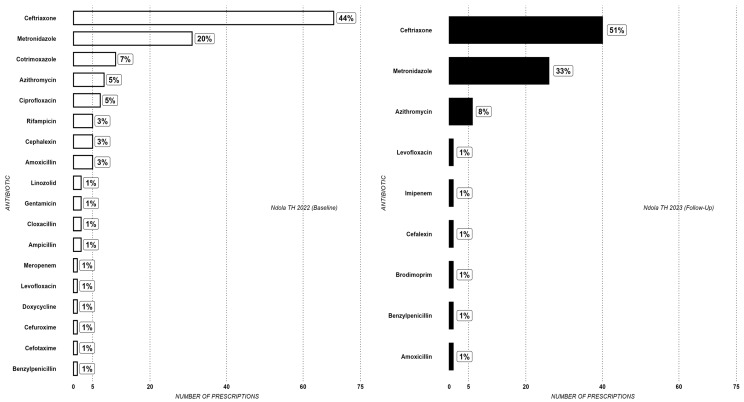
Antibiotic prescribing rate at Ndola TH at baseline (2022) and follow-up (2023).

In Figure 2, while ceftriaxone and metronidazole also accounted for the highest proportions at NTH, an increase in their combined and individual proportions was observed in 2023 compared to 2022.

**Figure 3:**
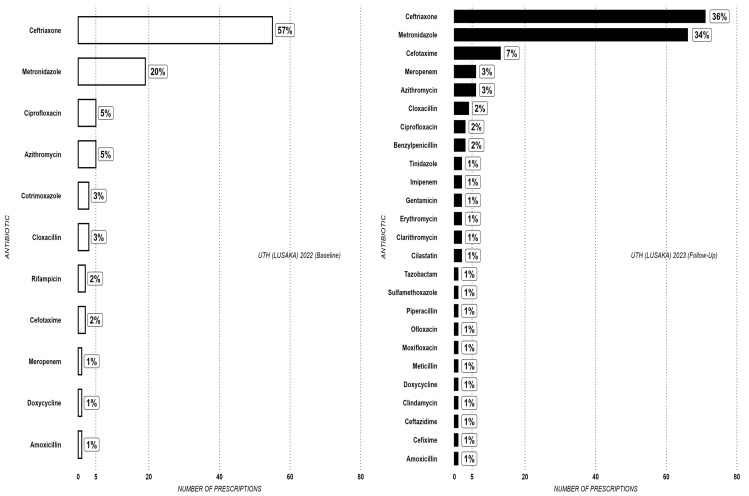
Antibiotic prescribing rate at UTH (Lusaka) at baseline (2022) and follow-up (2023).

Figure 3 illustrates that UTH also reported that ceftriaxone and metronidazole were the most prescribed antibiotics. However, whereas a direct relationship was noted between the proportions of the two antibiotics at the other two sites, the data from UTH exhibited an inverse relationship between the proportions of ceftriaxone and metronidazole. In this regard, there was a noticeable drop in the use of ceftriaxone, from 57% to 36%, while metronidazole increased from 20% to 34%. Overall, the average prescribing of ceftriaxone was 48% in 2022 and 38% in 2023, demonstrating a 10% reduction (Figure 3).

## 3. Discussion

To the best of our knowledge, this was the first PPS assessing the impact of AMS programs on antibiotic use and prescription patterns in Zambia. Overall, this study found a 75% (average of 1.27 number of antibiotics) prevalence of antibiotic use. Before the AMS intervention, 81% (average of 1.38 antibiotics) of the in-patients were on at least one antibiotic, with the majority being on ceftriaxone, indicating a high use of the Watch category of antibiotics. The prevalence of antibiotic use decreased to 71% (average of 1.21 number of antibiotics) during the AMS intervention, with most in-patients receiving ceftriaxone, followed by metronidazole, cefotaxime, and azithromycin. Overall, there were equal proportions of Access (50%) and Watch (50%) categories of antibiotics prescribed before the AMS intervention. On the other hand, following the implementation of AMS at the hospitals, 43% of the prescribed antibiotics were from the Access group while 57% were from the Watch group, indicating an increase in the use of Watch antibiotics. Additionally, compliance with the STG was 42% pre-intervention (2022) compared to 45% during the AMS intervention (2023). The results indicate an early positive impact of the AMS intervention, which would likely improve as the continued implementation of the AMS program leads to behavior change in the use of antibiotics and prescription patterns.

The pre- and post-intervention prevalence of antibiotic use in our study was higher than that found in an earlier study in Zambia [90]. The prevalence of antibiotic use found in our study was higher than the prevalence reported in 2017 in Nigeria (69.7%) [108], Botswana (70.6%) [109], and Sierra Leone (73.3%) [110], but similar to the 75% and 77.6% reported in Pakistan [82,111], 78% in Bangladesh [76], 78.9% in India [112], 80.6% in 2022 in Nigeria [113], and 82.9% in Benin [69]. The prevalence of antibiotic use reported in our study was higher than that reported in other studies, which ranged from 33.8% to 68% [77,80,85,107,114,115,116,117,118,119,120,121]. The high prescribing and use of antibiotics in hospitals require urgent attention and interventions to prevent the emergence and spread of AMR.

AMS interventions promote the rational use of antibiotics through improved awareness and knowledge of antibiotic use and AMR, reduction in antibiotic prescribing and use, and adherence to treatment guidelines [122,123,124,125], which results in a decline in antibiotic use. The decline in antibiotic use prevalence following the AMS intervention in our study demonstrates the impact AMS interventions can have on antibiotic use, which has been supported by studies elsewhere. For instance, the use of antibiotics was reduced due to the educational activities targeting healthcare workers in hospitals in Saudi Arabia, Ghana, and Uganda [126,127,128,129]. A recent study in Germany reported no decline in the use of antibiotics, although the total consumption reduced three years after the introduction of AMS programs in a University Teaching Hospital Emergency Department [130]. Another study in Ghana reported a decline in the use of antibiotics from 65% to 59.7% following the introduction of AMS programs in a District Hospital [127]. Further, similar to our findings, a study in the United Arab Emirates (UAE) reported a 6% decrease in the proportion of patients on antibiotics following the introduction of AMS interventions [131]. Therefore, strengthening a robust, coordinated, multifaceted, and sustained multidisciplinary AMS intervention has the potential to promote the rational use of antibiotics [131,132]. However, if not addressed, negative behaviors among prescribers may negatively impact the effects of AMS programs in healthcare facilities [130]. Hence, the promotion of behavior change towards rational prescribing and use of antibiotics is critical [133,134,135,136].

Ceftriaxone was the most prescribed and used antibiotic across the three tertiary hospitals pre-AMS and during the AMS intervention implementation. Other highly prescribed antibiotics included ciprofloxacin and metronidazole pre-intervention and metronidazole and azithromycin post-intervention. However, our study indicated a reduction in the use of ceftriaxone from 48% in the pre-AMS intervention to 38% in the post-AMS intervention, demonstrating a 10% decrease in the use of ceftriaxone. Similarly, the consumption of other antibiotics was reduced in the post-intervention compared to the pre-AMS intervention. The overuse of ceftriaxone in Zambian hospitals was similar to what has been reported in earlier studies [86,88,90,119,121,137,138]. Ceftriaxone is a Watch antibiotic with a low genetic barrier to resistance; therefore, if used irrationally, most pathogens would become resistant to it [139]. Additionally, being a third-generation cephalosporin, its overuse may lead to the emergence of extended-spectrum beta-lactamases (ESBLs) [140,141,142,143]. A study conducted in Malawi also revealed a 26.5% reduction in the use of ceftriaxone from 80.1% pre-AMS intervention to 53.6% post-AMS intervention [144]. Similarly, a study in Germany showed a reduction in the use of cephalosporins and fluoroquinolones after the implementation of AMS programs and an increase in the use of narrow-spectrum antibiotics [130]. An Italian study also reported a decrease in the use of ceftriaxone from 15.3% to 6% after the introduction of an effective AMS program [132]. A study in Jordan reported a reduction in the use of broad-spectrum antibiotics even though this was not sustained when the AMS program was halted, indicating the need for a sustained AMS program in the hospitals [145].

The present study found a modest increase (3%) in compliance with the national STGs from 42% in 2022 to 45% in 2023. The compliance level in our study is similar, though less than the compliance level (50.4%) in a study conducted in Malaysia [120]. Our findings are similar to those reported in a study in the UAE, which showed that instigation of AMS programs improved adherence to the treatment guidelines from 59% in 2019 to 67% in 2022 [131]. In the United States, a study reported that some low-cost interventions improved adherence to treatment guidelines in prescriptions for acute respiratory tract infections [146]. It is noteworthy that interventions to improve adherence to the treatment guidelines should be coupled with behavior change to promote the rational use of antibiotics [147]. Therefore, well-implemented AMR interventions lead to improved adherence to the treatment guidelines [45,148,149].

Our study revealed equal prescription of the Access and Watch categories pre-intervention (2022) and higher prescription of the Watch (57%) compared to the Access (43%) post-intervention (See Tables S1 and S2). In both assessments, there were no prescribed Reserve drugs. Our findings indicate an increase in the use of Watch antibiotics (mostly ceftriaxone), which is not in line with the WHO AWaRe framework recommendation that 60% of the prescribed antibiotics should be from the Access group [57,58,61]. This could be a result of a lack of information and implementation of the WHO AWaRe framework. Deviations in adherence to the WHO AWaRe classification of antibiotics have been reported in other studies [72,76,87,90,150]. The high use of Watch-group antibiotics was also reported in a PPS conducted in Japan, where 58.4% of the prescribed antibiotics were from the Watch group [151]. On the other hand, our findings are in line with a study performed in Ghana, Uganda, and Zambia in which Reserve antibiotics were not prescribed in the surveyed hospitals [107]. On the contrary, a study in Ghana reported that after the introduction of AMS programs in hospitals, the use of Access antibiotics increased from 40% to 50% while the Watch group reduced from 60% to 50%, indicating effective implementation of AMS programs [127]. Therefore, there is a need for continuous education and implementation of AMS programs in Zambia to improve and maintain the rational use of antibiotics.

We are aware of the limitations of our study. Our study was conducted in three tertiary hospitals in three out of the ten provinces in Zambia; hence, the generalization of our findings must be performed with caution. Secondly, the implementation period to detect the actual impact of AMS interventions on antibiotic use was too short. However, our findings show the early gains of introducing and implementing AMR programs in healthcare facilities.

## 4. Materials and Methods

### 4.1. Study Design, Setting, and Population

**Figure 4:**
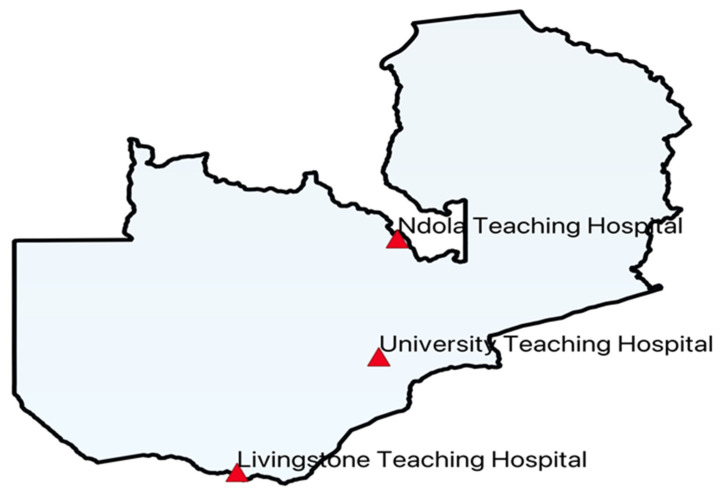
Map of Zambia showing the location of the surveyed hospitals.

This was a before-and-after study of the effect of the AMS program on antibiotic use and prescribing patterns. The study was part of the 3-year AMS demonstration project funded by ICARS. The objective of the project is to optimize the use of antibiotics in the treatment of bloodstream infections (BSIs) and urinary tract infections (UTIs) in various health-sector settings in Zambia. We applied the WHO PPS methodology at baseline in August 2022, and a follow-on PPS followed by another one in October 2023 at the three tertiary hospitals, namely Ndola Teaching Hospital (NTH, Copperbelt Province, bed capacity of 851 and a medical ward capacity of 203), Livingstone Teaching Hospital (LTH, Southern Province, bed capacity of 242 and a medical ward capacity of 91), and University Teaching Hospitals (UTH, Lusaka Province, bed capacity of 830 and a medical ward capacity of 380). The survey sites are shown in Figure 4. The survey population included all adult in-patients admitted to the surgical and medical wards before 08:00 h on the day of the survey. All patients who were admitted to the ward by 08:00 h on the day of the survey were eligible. Antibiotics included antibacterials for oral and systemic use and intestinal anti-infective and antiprotozoal agents. Antibiotics used for HIV prophylaxis, such as co-trimoxazole, anti-tuberculous treatment (ATT), and antifungals, were excluded. Also, outpatients and daytime admissions for ambulatory patients for procedures such as endoscopy or renal dialysis and those patients on antibiotics for topical use were excluded from the survey.

### 4.2. Sample Size Estimation and Sampling Criteria

We selected the study sites using purposive sampling methods. This was because the three institutions were planning to strengthen AMS programs and develop deliberate interventions to improve the rational use of antibiotics. We selected all the medical and surgical patients that met the inclusion criteria. Since the targeted medical and surgical wards had bed capacities of less than 500, we included all the in-patients who met the inclusion criteria. Our sample size estimation is in line with the WHO PPS methodology recommendations, which state that for a bed capacity of less than 500, all patients that meet the inclusion criteria on the day of the PPS must be included in the study [70].

### 4.3. Data Collection

We used the WHO PPS tool to collect the data [70]. Data were collected through REDCap version 9.1.15, a web-based platform [152]. Access to this application was provided to all investigation teams, which included the pharmacists, laboratory scientists, nurses, and physicians, via email and uploaded onto hand-held devices.

Data were collected from patients’ medical files who were hospitalized before 08:00 h on the day of the surveys in the medical and surgical wards. Data were collected using two forms: a ward form used to record the denominators (number of beds and number of admitted patients before or at 08:00 h on the day of the PPS) and a patient form used to record detailed antimicrobial prescription (type, dose, administration route, indication, and diagnosis) for those patients who had received at least one antimicrobial before the day of the PPS. The entire data collection did not exceed three days in a single hospital or one day in each ward. Patients’ recorded data were anonymized.

### 4.4. Study Variables

The WHO PPS tool used standardized codes for differentiating wards in the hospitals [70]. Variables at the hospital level included the hospital name and the level of care. Further, ward-level variables included patient census data, patient name, code, and specialty of the wards. At the patient level, variables were divided into two sections, with the first section containing variables for all included in-patients and the second for only those patients on antimicrobial therapy during admission. The variables collected included demographic details such as age, gender, and admission for the same condition or a related complication arising from the previous admission [70].

Other variables included prescriber designations, reasons for prescription of antibiotics (treatment or prophylaxis), and if for prophylaxis, whether it was for medical or surgical prophylaxis. In addition, the duration of surgical prophylaxis is one dose, either one day or longer, admission diagnosis, and type of infection [70].

We collected data on antibiotics prescribed, whether prescribed on the drug sheet, whether by INN (International Nonproprietary Name) and whether it was from the Zambia STGs and Essential Medicines List (EML) [153,154], indication, start/stop date, dose, and frequency of administration.

The treatment of infections was classified into community-acquired infections (CAIs) and healthcare-associated infections (HAIs) as well as home-based care infections (HBCIs). Infections were considered CAIs if patients presented with infections or symptoms occurred <48 h from admission, HAIs if symptoms appeared >48 h after hospital admission based on chart review, and HBCIs if a terminally ill patient was transferred in from a long-term care facility due to an infection.

We categorized all the prescribed antibiotics according to the WHO AWaRe classification of antibiotics [55,56,57,61,64,155]. The results of the WHO AWaRe classification are shown in Tables S1 (for the year 2022) and Table S2 (for the year 2023).

### 4.5. Training and Supervision

Before the PPS, a two-day training was conducted at the University Teaching Hospital in Lusaka for the data collectors, comprising pharmacists, medical doctors, and an expert in health surveys from each of the three hospitals. The training was based on the PPS method, the study’s aims, the characteristics and contents of the questionnaires, and the procedures for data collection processes. Each team at the three hospitals was under the direct supervision of at least one principal investigator (pharmacist) during the PPS in the hospitals.

Data validation was performed by reviewing records to identify missing information and duplications. Records with incomplete information and duplicate entries were rectified after discussing with the duty physicians and nurses. Data were also evaluated for consistency and to ensure reliability using a pre-tested standardized questionnaire.

### 4.6. Antimicrobial Stewardship Interventions

Situational analyses and baseline PPS were conducted at the three facilities. Several gaps were identified, and interventions were put in place for a period of 15 months (from August 2022 to October 2023).

The major intervention was the setting up of AMS programs in facilities such as NTH and LTH where the program was non-existent. The AMS program was in place at UTH but was inactive and so needed strengthening. Other interventions that were put in place include the following:

- Baseline assessment of knowledge, attitudes, and practices on AMS/AMR of healthcare workers at 3 institutions.
- Baseline training and education in AMS for the AMS teams and the other healthcare workers (HCWs) at the facilities because inadequate knowledge of AMS was the biggest gap that was identified.
- The AMS teams conducted quarterly continuous medical education on AMS for the HCWs.
- Awareness and promotion of the use of STGs among the prescribers through hospital clinical meetings.
- Introduction of antibiotic prescription charts with automatic stop orders.
- Prospective multidisciplinary (physicians, pharmacists, nurses, and microbiologists) AMS ward rounds with real-time feedback to the prescribers once every week during the study time.

### 4.7. Data Analysis

The data collected through REDCap version 9.1.15 were exported to Microsoft (MS) Excel 2010. Preliminary cleaning, which included the renaming of variables of interest and ensuring consistency of data types for the concerned variables, was performed in MS Excel. The data were then imported into R version 4.4.0 for data wrangling using the dplyr package, descriptive data analysis and hypothesis testing using the gtsummary package, and data visualization using the ggplot2 package. Unless otherwise stated, a significance level of 5% was assumed.

All categorical variables were described using frequencies and percentages. This included estimations of both the proportion of prescriptions that complied with the national STG and the prescribing rates of respective antibiotics. Age being a skewed variable, it was summarized using the median and interquartile range (IQR). On the other hand, since the number of prescribed antibiotics followed a normal distribution, it was summarized using the mean and standard deviation (SD).

Assuming expected values for all cells in a cross-tabulation were greater or equal to five, the relationship between each categorical variable and the year in which the survey was conducted was tested using the chi-square test for independence. Where the aforementioned assumption was violated, Fisher’s exact test was used instead. The Wilcoxon rank sum test was used to test the relationship between each numerical variable and the year in which the survey was conducted.

## 5. Conclusions

This study revealed a high rate of antibiotic usage at three tertiary hospitals in Zambia, particularly preceding the Antimicrobial Stewardship (AMS) intervention. Notably, the AMS intervention yielded a significant reduction in antibiotic utilization (*p*-value < 0.001), specifically minimizing the prescription and administration of ceftriaxone. Furthermore, the intervention enhanced adherence to Zambia’s national Standard Treatment Guidelines (STG). However, sustaining these gains necessitates continuous implementation of AMS programs. Therefore, it is imperative to establish and maintain robust AMS initiatives across all hospitals in Zambia. This will be crucial in combating the growing threat of Antimicrobial Resistance (AMR), ultimately ensuring the long-term effectiveness of antibiotics and promoting public health.

## Figures and Tables

**Table 1:** Demographic characteristics at baseline (2022) and follow-up (2023) point prevalence surveys.

	All Sites				Livingstone TH				Ndola TH				UTH (Lusaka)			
VARIABLES	Overall N = 437 ^1^	2022 N = 172 ^1^	2023 N = 265 ^1^	Pr ^2^	Overall N = 64 ^1^	2022 N = 21 ^1^	2023 N = 43 ^1^	Pr ^2^	Overall N = 148 ^1^	2022 N = 74 ^1^	2023 N = 74 ^1^	Pr ^2^	Overall N = 223 ^1^	2022 N = 75 ^1^	2023 N = 148 ^1^	Pr ^2^
Age of patient in years				0.4				0.8				0.3				0.7
Median (Q1, Q3)	44 (30, 58)	45 (31, 60)	44 (29, 56)		40 (25, 53)	40 (23, 53)	41 (26, 52)		48 (32, 65)	50 (35, 65)	46 (30, 65)		43 (29, 55)	43 (28, 54)	44 (32, 56)	
*	1	2	9		1		1		4		4		4		4	
Gender of patient				>0.9				0.12				0.8				0.5
Female	199 (46%)	78 (47%)	121 (46%)		27 (42%)	6 (29%)	21 (49%)		70 (48%)	36 (49%)	34 (47%)		102 (47%)	36 (50%)	66 (45%)	
Male	230 (54%)	89 (53%)	141 (54%)		37 (58%)	15 (71%)	22 (51%)		77 (52%)	38 (51%)	39 (53%)		116 (53%)	36 (50%)	80 (55%)	
*	8	5	3						1				5	3	2	

^1^ n (%); * missing values; Q1: quartile 1; Q3: quartile 3; ^2^ Wilcoxon rank sum tests; Pearson’s chi-squared test.

**Table 2:** Prescribing behavior at baseline (2022) and follow-up (2023).

	All Sites				Livingstone TH				Ndola TH				UTH (Lusaka)			
VARIABLES	Overall N = 427 ^1^	2022 N = 172 ^1^	2023 N = 265 ^1^	Pr ^2^	Overall N = 64 ^1^	2022 N = 21 ^1^	2023 N = 43 ^1^	Pr ^2^	Overall N = 148 ^1^	2022 N = 74 ^1^	2023 N = 74 ^1^	Pr ^2^	Overall N = 223 ^1^	2022 N = 75 ^1^	2023 N = 148 ^1^	Pr ^2^
Compliance with STGs				<0.001				<0.001				<0.001				<0.001
Compliance	137 (44%)	59 (42%)	78 (45%)		21 (50%)	0	21 (88%)		47 (44%)	37 (59%)	10 (22%)		69 (42%)	22 (37%)	47 (46%)	
Insufficient Information	25 (8.0%)	6 (4.3%)	19 (11%)		1 (2.4%)	0	1 (4.2%)		20 (19%)	2 (3.2%)	18 (40%)		4 (2.5%)	4 (6.7%)	0	
Non-Compliance	83 (27%)	21 (15%)	62 (36%)		12 (29%)	11 (61%)	1 (4.2%)		10 (9.3%)	2 (3.2%)	8 (18%)		61 (37%)	8 (13%)	53 (51%)	
Not Assessable	68 (22%)	55 (39%)	13 (7.6%)		8 (19%)	7 (39%)	1 (4.2%)		31 (29%)	22 (35%)	9 (20%)		29 (18%)	26 (43%)	3 (2.9%)	
*	124	31	93		22	3	19		40	11	29		60	15	45	
Patients categories				0.001				0.11				0.001				0.083
No antibiotics prescribed	110 (25%)	32 (19%)	78 (29%)		17 (27%)	2 (9.5%)	15 (35%)		38 (26%)	10 (14%)	28 (38%)		53 (24%)	18 (24%)	35 (24%)	
1 Antibiotic prescribed	126 (29%)	63 (37%)	63 (24%)		17 (27%)	6 (29%)	11 (26%)		40 (27%)	26 (35%)	14 (19%)		69 (31%)	31 (41%)	38 (26%)	
2 Antibiotics prescribed	175 (40%)	60 (35%)	115 (43%)		25 (39%)	10 (48%)	15 (35%)		58 (39%)	26 (35%)	32 (43%)		92 (41%)	24 (32%)	68 (46%)	
3 Antibiotics prescribed	23 (5.3%)	14 (8.1%)	9 (3.4%)		4 (6.3%)	2 (9.5%)	2 (4.7%)		10 (6.8%)	10 (14%)	0		9 (4.0%)	2 (2.7%)	7 (4.7%)	
4 Antibiotics prescribed	3 (0.7%)	3 (1.7%)	0		1 (1.6%)	1 (4.8%)	0		2 (1.4%)	2 (2.7%)	0		0	0	0	
Number prescribed				0.2				0.026				0.004				0.10
Mean (SD)	1.27 (0.92)	1.38 (0.94)	1.21 (0.91)		1.30 (0.99)	1.71 (0.96)	1.09 (0.95)		1.31 (0.97)	1.57 (0.98)	1.05 (0.90)		1.26 (0.87)	1.13 (0.81)	1.32 (0.89)	

^1^ n (%); * missing values; ^2^ Pearson’s chi-squared tests; Fisher’s exact test; Wilcoxon rank sum test.

## Data Availability

Data are available and can be shared on request from the corresponding author.

## Supplementary Materials

The following supporting information can be downloaded at https://www.mdpi.com/article/10.3390/antibiotics14030284/s1, Table S1: Antibiotic Prescribing Patterns by AWaRe Classification in 2022; Table S2: Antibiotic Prescribing Patterns by AWaRe Classification in 2023.
