# Supplementary material for: Antimicrobial Stewardship Impact on Antibiotic Use in Three Tertiary Hospitals in Zambia: A Comparative Point Prevalence Survey
Source: Antibiotics (Basel). 2025 Mar 10;14(3):284. doi: 10.3390/antibiotics14030284 (PMC11939251; doi:10.3390/antibiotics14030284)
Supplement: Supplementary file 1 [file antibiotics-14-00284-s001.zip › antibiotics-3459784-supplementary.pdf]

Article

Antimicrobial Stewardship Impact on Antibiotic Use in Three Tertiary Hospitals in Zambia: A Comparative Point Prevalence Survey

In 2022, the PPS revealed that the most prescribed antibiotic among the three tertiary hospitals was ceftriaxone (50%), with UTH and NTH having had the highest number of prescriptions of ceftriaxone at 56% and 47%, respectively. Other highly prescribed antibiotics included metronidazole (20%), azithromycin (5.9%), and ciprofloxacin (5.5%). Fifty percent (50%, 8/16) of the antibiotics prescribed for in-patients were from the Access group while the other 50% (8/16) were from the Watch group. Notably, no Reserve antibiotics were prescribed in the three hospitals during the year 2022 (Table S1).

Table S1. Prescribing patterns of antibiotics according to the AWaRe classification of antibiotics, 2022.

DRUGS	Overall, N = 237	LTH, N = 36	NTH, N = 116	UTH, N = 85	AWaRe classification
Name of antibiotic	n (%)	n (%)	n (%)	n (%)	
Amoxicillin	6 (2.5)	2 (5.6)	3 (2.6)	1 (1.2)	Access
Azithromycin	14 (5.9)	2 (5.6)	7 (6.0)	5 (5.9)	Watch
Benzylpenicillin	1 (0.4)	1 (2.8)	0	0	Access
Cefotaxime	4 (1.7)	1 (2.8)	1 (0.9)	2 (2.4)	Watch
Ceftriaxone	118 (50)	16 (44)	54 (47)	48 (56)	Watch
Cefuroxime	1 (0.4)	0	1 (0.9)	0	Watch
Cephalexin	3 (1.3)	0	3 (2.6)	0	Access
Ciprofloxacin	13 (5.5)	3 (8.3)	7 (6.0)	3 (3.5)	Watch
Cloxacillin	5 (2.1)	0	2 (1.7)	3 (3.5)	Access
Cotrimoxazole	12 (5.1)	1 (2.8)	8 (6.9)	3 (3.5)	Access
Doxycycline	2 (0.8)	0	1 (0.9)	1 (1.2)	Access
Gentamicin	1 (0.4)	0	1 (0.9)	0	Access
Levofloxacin	1 (0.4)	0	1 (0.9)	0	Watch
Meropenem	2 (0.8)	0	1 (0.9)	1 (1.2)	Watch
Metronidazole	47 (20)	10 (28)	21 (18)	16 (19)	Access
Rifampicin	7 (3.0)	0	5 (4.3)	2 (2.4)	Watch

In the 2023, ceftriaxone (39%) was the most prescribed antibiotic in the three tertiary hospitals, with NTH and UTH having the highest number of ceftriaxone prescriptions at 51% and 36%, respectively. Metronidazole (34%), was the second most prescribed antibiotic, with equal proportions being prescribed at both LTH and UTH. Cefotaxime (6.7%) was only prescribed at UTH, which was followed by azithromycin (3.8%). UTH had the highest number of antibiotics prescribed, 60.9% (195/320). Most prescribed antibiotics 57% (17/30) were from the Watch group, followed by the Access class, 43% (13/30). Notably, no *R*eserve antibiotics were prescribed in the three hospitals during the year 2023 (Table S2).

Table S2. Prescribing patterns of antibiotics according to the AWaRe classification of antibiotics, 2023.

Drugs<br>Name of antibiotic	Overall,<br>N = 320<br>n (%)	LTH,<br>N = 47<br>n (%)	NTH,<br>N = 78<br>n (%)	UTH,<br>N = 195<br>n (%)	AWaRe classification
Amoxicillin	3 (0.9)	1 (2.1)	1 (1.3)	1 (0.5)	Access
Azithromycin	12 (3.8)	0	6 (7.7)	6 (3.1)	Watch
Benzylpenicillin	4 (1.3)	0	1 (1.3)	3 (1.5)	Access
Brodimoprim	1 (0.3)	0	1 (1.3)	0	Access
Cefalexin	2 (0.6)	1 (2.1)	1 (1.3)	0	Access
Cefixime	1 (0.3)	0	0	1 (0.5)	Watch
Cefotaxime	13 (4.1)	0	0	13 (6.7)	Watch
Ceftazidime	1 (0.3)	0	0	1 (0.5)	Watch
Ceftizoxime	1 (0.3)	1 (2.1)	0	0	Watch
Ceftriaxone	124 (39)	13 (28)	40 (51)	71 (36)	Watch
Cilastatin	2 (0.6)	0	0	2 (1.0)	Watch
Ciprofloxacin	7 (2.2)	4 (8.5)	0	3 (1.5)	Watch
Clarithromycin	2 (0.6)	0	0	2 (1.0)	Watch
Clindamycin	1 (0.3)	0	0	1 (0.5)	Access
Cloxacillin	5 (1.6)	1 (2.1)	0	4 (2.1)	Access
Doxycycline	2 (0.6)	1 (2.1)	0	1 (0.5)	Access
Erythromycin	2 (0.6)	0	0	2 (1.0)	Watch
Gentamicin	2 (0.6)	0	0	2 (1.0)	Access
Imipenem	3 (0.9)	0	1 (1.3)	2 (1.0)	Watch
Levofloxacin	1 (0.3)	0	1 (1.3)	0	Watch
Meropenem	6 (1.9)	0	0	6 (3.1)	Watch
Meticillin	1 (0.3)	0	0	1 (0.5)	Access
Metronidazole	108 (34)	16 (34)	26 (33)	66 (34)	Access
Moxifloxacin	1 (0.3)	0	0	1 (0.5)	Watch
Ofloxacin	1 (0.3)	0	0	1 (0.5)	Watch
Piperacillin	1 (0.3)	0	0	1 (0.5)	Watch
Sulfamethoxazole	5 (1.6)	4 (8.5)	0	1 (0.5)	Access
Tazobactam	1 (0.3)	0	0	1 (0.5)	Watch
Tinidazole	2 (0.6)	0	0	2 (1.0)	Access
Trimethoprim	5 (1.6)	5 (11)	0	0	Access
